# Comparison between hypersensitivity reactions to cycles of modified FOLFOX6 and XELOX therapies in patients with colorectal cancer

**DOI:** 10.1007/s00280-017-3294-9

**Published:** 2017-04-08

**Authors:** Hideki Ohta, Takahiro Hayashi, Sumie Murai, Hideyo Shiouchi, Yosuke Ando, Satomi Kumazawa, Kaori Ito, Yoshiaki Ikeda, Hiroshi Matsuoka, Kotaro Maeda, Kenji Kawada, Kimio Yasuda, Shigeki Yamada

**Affiliations:** 1 Department of Clinical Pharmacy, Fujita Health University School of Medicine, 1-98 Dengakugakubo, Kutsukake-cho, Toyoake, 470-1192 Japan; 2 Department of Pharmacy, Fujita Health University Hospital, Toyoake, Japan; 3 College of Pharmacy, Kinjo Gakuin University, Nagoya, Japan; 4 Department of Surgery, Fujita Health University School of Medicine, Toyoake, Japan; 5 Department of Medical Oncology, Fujita Health University School of Medicine, Toyoake, Japan; 6 Department of Hematology, Fujita Health University School of Medicine, Toyoake, Japan

**Keywords:** Oxaliplatin, Hypersensitivity reactions, XELOX therapy, FOLFOX therapy, Colorectal cancer

## Abstract

**Purpose:**

Although hypersensitivity reactions (HSRs) to oxaliplatin (L-OHP) therapy are well-documented, few reports have compared different therapies in terms of HSR occurrence. In this study, we compared the frequency and pattern of HSRs to modified FOLFOX6 (mFOLFOX6; 5-fluorouracil, levofolinate calcium and L-OHP infusions) and XELOX (capecitabine and L-OHP) therapies, and sought to identify risk factors associated with HSRs.

**Methods:**

Patients who had received mFOLFOX6 or XELOX chemotherapeutic regimens for unresectable colon or rectal cancer or as adjuvant chemotherapy following colon cancer surgery between April 2012 and August 2015 were included. Potential correlation between treatment modalities (regimen, dosage and route of administration of L-OHP, and injection timing for dexamethasone administration) and HSRs was assessed.

**Results:**

Among the 240 patients included in the study, 136 had received mFOLFOX6 therapy and 104 had received XELOX therapy. Although the frequency of HSRs did not differ between the two groups, incidence of HSRs in the first cycle was higher in the XELOX therapy group. Treatment method or cumulative dosage was not identified as a risk factor for HSR; however, the incidence of ≥grade-2 HSR was higher in cases where the cumulative L-OHP dosage was ≥600 mg/m^2^ and in patients in whom dexamethasone was not co-infused with L-OHP.

**Conclusion:**

Although HSR rates were comparable among patients treated with mFOLFOX6 and XELOX, HSRs tended to occur more frequently during the first cycle of XELOX therapy as compared to that with mFOLFOX6 therapy. Our findings warrant careful assessment of ≥grade-2 HSRs in patients who are prescribed cumulative L-OHP dosages of ≥600 mg/m^2^.

## Introduction

In a previous trial on patients with advanced colorectal cancer [1], FOLFOX4 therapy [5-fluorouracil (5-FU), levofolinate calcium (LV) and oxaliplatin (L-OHP) infusions] was shown to be superior to conventional therapy (5-FU and LV infusions) in terms of progression-free survival and response rates. Based on these results, L-OHP is positioned as a key drug in the treatment of colorectal cancer. Further, modified FOLFOX6 (mFOLFOX6) and capecitabine plus L-OHP (XELOX) therapies are frequently used for these patients [2, 3].

Platinum-based compounds are in use as anticancer agents since the 1970s, and have been developed in the following order: cisplatin, carboplatin, and L-OHP. Despite their efficacy as anticancer agents, these drugs produce serious side effects and hypersensitivity reactions (HSRs). Although the type of HSR, incidence rate and time to occurrence tend to vary among platinum compounds [4], the ensuing details remain largely unknown. Reported rates of HSRs to L-OHP, in the absence of any countermeasures, range from 8.9 to 23.8% [5–17]. In a comparative study of three platinum-based compounds published in 2010, HSRs to L-OHP typically occurred after six treatment cycles [4]. Similarly, in a study of eight regimens, including FOLFOX and XELOX, the mean and median (range) numbers of treatment cycles for HSRs were 7.9 and 8 (4–12), respectively[15]. In contrast, in a study of more than six regimens, including FOLFOX4 and GEMOX (L-OHP+ gemcitabine) therapies, HSRs were reported at a median of 4.7 cycles [16]. However, because differing treatment regimens likely have differing HSR onset times, analyses of HSRs under conditions of mixed treatment methods are likely biassed.

Known risk factors for HSRs to L-OHP include female sex [16, 18], young age [16, 18], initial treatment with a platinum-based anticancer agent [16, 18] and repeated administration of L-OHP [11]. In a study of XELOX therapy by Yoshida et al. [19], the combination of dexamethasone (Dex; 6.6 mg) and L-OHP (a total of 13.2 mg Dex) reduced the incidence of HSRs to 4.1%; further, only grade-1 skin symptoms were observed with co-administration of Dex and L-OHP. This suggests that the amount or timing of steroid administration has a strong effect on HSR occurrence.

Here we report a retrospective comparison between the frequency and pattern of HSRs in patients treated with mFOLFOX6 and XELOX therapy, and identified risk factors for HSR.

## Materials and methods

### Subjects

Subjects included patients who received mFOLFOX6 or XELOX therapy as cancer chemotherapy for unresectable colon or rectal cancer, or as adjuvant chemotherapy following colon cancer surgery, at the Fujita Health University Hospital between April 2012 and August 2015. All patients who did not agree with the main purpose of the study were excluded. Patients who received mixed treatment with XELOX and mFOLFOX6 therapies, administered with pre-treatment steroids or anti-allergic drugs or have missing data due to a change of hospital or death during treatment were excluded.

### Investigations

This was a retrospective study based on patient data collected from electronic patient files available in the databases of Fujita Health University Hospital.

**Fig. 1:**
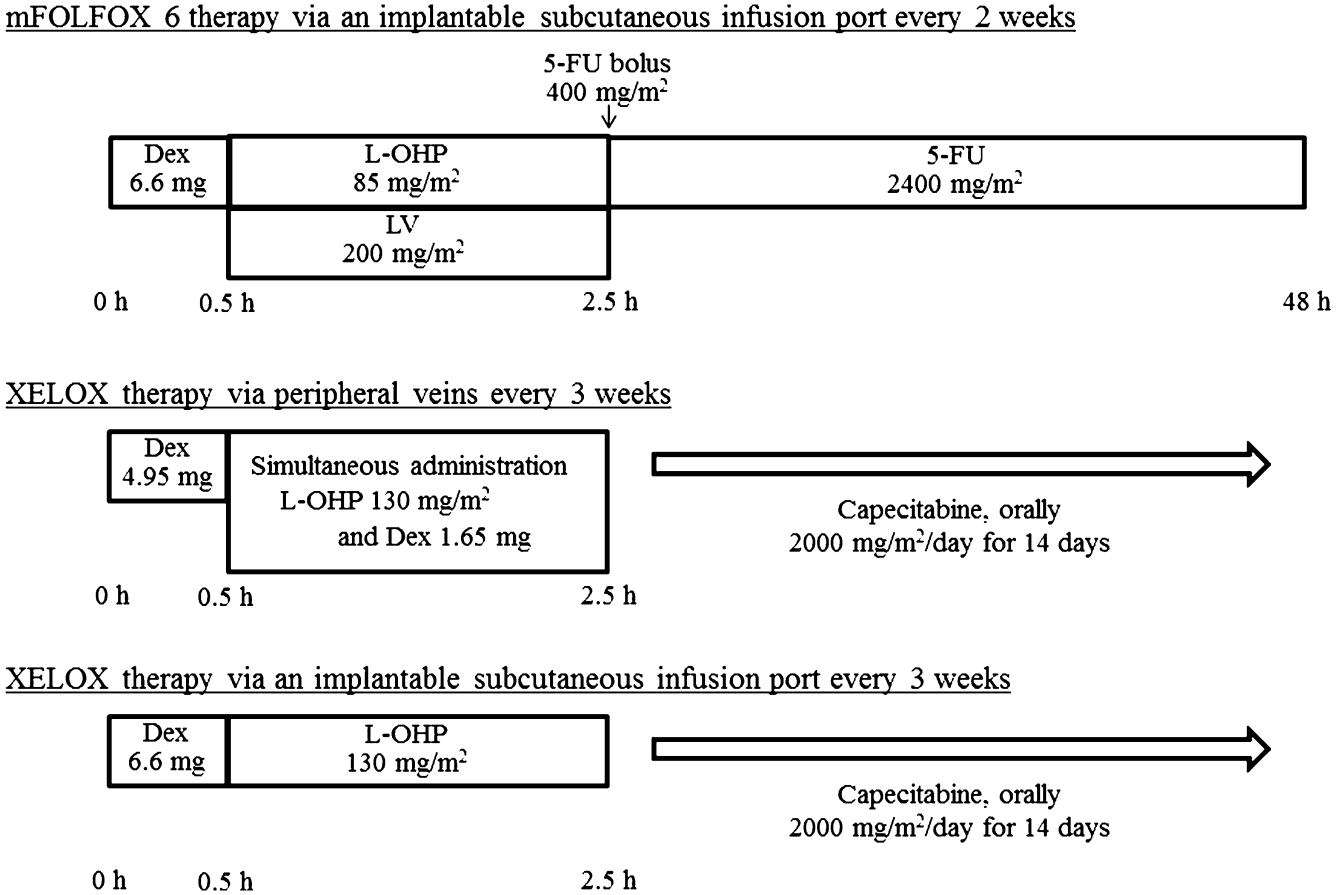
Chemotherapy regimens. *Dex* dexamethasone, *L-OHP* oxaliplatin, *LV* levofolinate, *5-FU* 5-fluorouracil

The mFOLFOX6 and XELOX regimens are illustrated in Fig. 1. Dosages of anticancer agents were titrated to patients’ condition.

Data on the following variables were extracted: age at the start of the study; sex; body height; body weight; body surface area; cancer stage and metastasis; detailed treatment history (past chemotherapy, history of platinum-based therapies, regimen used, dosage/interval/route of administration of L-OHP, timing and amount of Dex, pre-treatment with steroids/antihistamines/anti-inflammatory drugs); and allergy-related information. Regarding allergies, the following items were obtained: vital signs (body temperature, blood pressure, respiratory rate, etc.), whether steroids/antihistamines/anti-inflammatory drugs were used, drug type, administration route, the presence or absence of a response to treatment, whether there had been prophylactic treatment and the drug type, whether any treatment had required hospitalization for HSR, and whether the HSR was protracted. In addition, descriptions related to the following items were used: dermal and mucosal symptoms (rash, eczema, pruritus, rubefaction, edema, and tumefaction), respiratory symptoms (dyspnea, respiratory stenosis, stridor, and hypoxemia), cardiovascular symptoms (hypotension and disturbance of consciousness), persistent digestive symptoms (abdominal colic and vomiting), and other symptoms (chills, nasal discharge, articulation disorders, etc.). We also investigated whether there was a relationship between persistent digestive symptoms and adverse effects of anticancer agents. Blood biochemical parameters included serum levels of albumin, aspartate aminotransferase (AST), alanine aminotransferase (ALT), serum creatinine (Scr) and eGFR at the commencement of treatment. Initial (in the first course) and cumulative dosages of L-OHP were recorded. In the event of an allergic or anaphylactic episode, the cumulative dosages received till that time were used in the analysis. Information related to allergic predisposition included history of allergy to drugs, food and pollen, related skin disorders (atopic and contact dermatitis) and specific history of allergy to platinum including that to L-OHP, cisplatin or carboplatin.

### Assessment

HSR severity was determined according to the Common Terminology Criteria for Adverse Events (CTCAE) v4.0.

### Statistical analysis

Normally distributed variables are expressed as mean ± standard deviation (SD); non-normally distributed variables are expressed as median [interquartile range (IQR)]. Parametric and non-parametric pairwise comparisons were performed using *t* test and Mann–Whitney *U* test, respectively. Ratios were compared using Chi-squared test, and risk factors for allergy were identified on univariate analysis. Subsequently, factors with hazard rates of less than 20% were incorporated in the multivariate logistic regression model. Goodness of fit was tested using Hosmer–Lemeshow tests. All statistical analyses were performed using SPSS software Ver. 22.0 (IBM Corporation, Armonk, NY, USA). Differences and associations with *P* values <0.05 were considered statistically significant.

### Ethics

The study protocol was approved by the Epidemiology and Clinical Research Ethics Review Board at the Fujita Health University.

## Results

### Patients

A total of 335 patients were enrolled in the study. Among these, 25 had received mixed treatment with XELOX and mFOLFOX6 therapies; in 13 patients, pre-treatment steroids or anti-allergic drugs were administered; data pertaining to 57 patients were missing ostensibly due to change of hospital or death during treatment. The remaining 240 patients included 136 patients in the mFOLFOX6 therapy group and 104 in the XELOX therapy group. No significant differences in demographic parameters were identified between the study groups at the start of the treatment (Table 1). However, differences were observed with respect to the dosage and route of administration of L-OHP and Dex and that of panitumumab administration (Table 1). At the start of the treatment, patients in the mFOLFOX therapy group had lower albumin levels and higher AST levels than those in the XELOX therapy group (Table 1).

**Table 1:** Patient characteristics

	mFOLFOX6 therapy (*n* = 136)	XELOX therapy (*n* = 104)	*P* value
Age (years)	66.0 (58.0–72.3)	65.5 (55.8–70.0)	0.067
Sex (male, female)	56, 80	51, 53	0.23
Body surface area (m^2^)	1.56 (1.42–1.69)	1.59 (1.45–1.72)	0.48
Skin disease career (%)	5.1	5.8	0.83
History of allergy to platinum (%)	27.9	28.8	0.88
History of treatment with L-OHP (%)	2.2	2.9	0.93
Medical history with L-OHP(%)	20.6	21.2	0.91
Stage (%)			
1	0	0	0.32
2	2.9	2.9	
3	17.6	27.9	
4	79.4	69.2	
Metastasis (%)	79.4	69.2	0.071
L-OHP			
By infusion port (%)	100	26.0	<0.001
Dosage (mg/m^2^/cycle)	82.2 (77.2–84.5)	123.0 (109.4–127.0)	<0.001
Cumulative dosage (mg/m^2^)	609.1 (417.1–919.1)	651.3 (454.4–942.1)	0.55
Cumulative dosage (mg)	915.0 (627.5–1469.5)	1000.0 (625.2–1502.9)	0.60
Monoclonal antibody			
Bmab (%)	42.6	41.3	0.92
Pmab (%)	11.0	0	0.001
Steroid			
Dex dosage (mg/cycle)	8.06 ± 0.72	7.93 ± 0.44	0.17
Co-administration with L-OHP (%)	0	74.0	0.001
Pre-medication with steroid except Dex (%)	10.3	16.3	0.17
Laboratory data			
Albumin (g/dL)	3.8(3.2–4.1)	4.0 (3.7–4.3)	0.002
AST (IU/L)	23.0 (17.8–34.0)	21.0 (17.0–28.0)	0.047
ALT (IU/L)	19.0 (12.0–30.0)	17.5 (12.0–26.0)	0.50
Scr (mg/dL)	0.70 (0.61–0.88)	0.71 (0.58–0.83)	0.45
eGFR (mL/min/1.73 m^2^)	75.2 (63.0–89.4)	78.2 (66.2–93.1)	0.30

*L-OHP* oxaliplatin, *Bmab* bevacizumab, *Pmab* panitumumab, *Dex* dexamethasone, *ASL* aspartate aminotransferase, *ALT* alanine aminotransferase, *Scr* serum creatinine, *eGFR* estimate glomerular filtration rate

### Risk factors for HSR

Potential risk factors for HSRs are presented in Table 2. Although treatment methods, cumulative L-OHP dosages, administration of monoclonal antibodies, concurrent steroid and L-OHP treatments, albumin and AST levels were not related to HSR, a body surface area of ≥1.57 m^2^ was significantly associated with a higher risk of HSR.

**Table 2:** Risk factors for hypersensitivity reaction

	Univariate analysis		Multivariate analysis	
	Odds ratio (95% CI)	*P* value	Odds ratio (95% CI)	*P* value
Age (years)	1.03 (0.99–1.06)	0.13	1.03 (0.99–1.06)	0.15
Female	0.65 (0.32–1.32)	0.24		
Body surface area ≥1.57 m^2^	1.79 (0.89–3.60)	0.11	2.18 (1.02–4.65)	0.045
Skin disease career	0.41 (0.052–3.28)	0.40		
Allergic history	0.72 (0.32–1.61)	0.43		
Platinum allergic history	5.50 (1.07–28.33)	0.042	4.97 (0.82–30.06)	0.081
Medical history with L-OHP	1.91 (0.89–4.10)	0.10	1.58 (0.64–3.94)	0.33
Metastasis	2.02 (0.80–5.09)	0.14	1.38 (0.50–3.79)	0.54
mFOLFOX6 therapy	0.68 (0.34–1.36)	0.28		
L-OHP dosage (mg/m^2^/cycle)	1.00 (0.99–1.02)	0.60		
L-OHP cumulative dosage (mg/m^2^)	1.00 (1.00–1.00)	0.29		
L-OHP cumulative dosage (mg)	1.00 (1.00–1.00)	0.16	1.00 (1.00–1.00)	0.61
Bmab	1.77 (0.89–3.52)	0.11	1.64 (0.77–3.49)	0.20
Pmab	1.31 (0.35–4.89)	0.69		
Steroid administered with L-OHP at the same time	0.80 (0.38–1.71)	0.57		
Dex dosage (mg/cycle)	1.00 (0.55–1.81)	1.00		
Pre-medication with steroid except Dex	1.28 (0.49–3.36)	0.62		
Albumin (g/dL)	0.77 (0.46–1.28)	0.30		
AST (IU/L)	1.00 (1.00–1.01)	0.12	1.00 (1.00–1.01)	0.59
ALT (IU/L)	1.01 (1.00–1.01)	0.14	1.00 (1.00–1.01)	0.42
Scr (mg/dL)	1.13 (0.23–5.61)	0.89		
eGFR (mL/min/1.73 m^2^)	1.00 (0.98–1.01)	0.62		

Risk factors are analysed with multivariable logistic regression models

*L-OHP* oxaliplatin, *Bmab* bevacizumab, *Pmab* panitumumab, *Dex* dexamethasone, *AST* aspartate aminotransferase, *ALT* alanine aminotransferase, *Scr* creatinine, *eGFR* estimated glomerular filtration rate

Hosmer–Lemeshow test, *P* = 0.62

### Comparisons of HSRs

The incidence of HSR in the mFOLFOX and XELOX therapy groups were 14.0 and 19.2%, respectively; no significant between-group difference was observed in this respect (*P* = 0.27).

No significant between-group difference was observed with respect to the severity of HSR (Table 3). However, the number of treatment cycles, dosages and routes of L-OHP administration differed between the groups (Table 3). No significant differences in cumulative dosages (mg/m^2^ or mg) were observed.

**Table 3:** Patients with hypersensitivity reactions

	mFOLFOX6 therapy (*n* = 19)	XELOX therapy (*n* = 20)	*P* value
HSR grade (%)			0.20
1	21.1	40.0	
2	73.7	40.0	
3	5.3	20.0	
4≤	0	0	
L-OHP			
By infusion port (%)	100	45.0	<0.001
Dosage (mg/m^2^/cycle)	82.2 (77.2–84.5)	123.0 (109.4–127.0)	<0.001
Cumulative dosage (mg/m^2^)	628.6 (471.4–1018.9)	599.5 (127.5–873.5)	0.32
Cumulative dosage (mg)	956.0 (720.0–1670.0)	990.0 (222.5–1570.0)	0.55
Number of cycles	10.0 (6.0–14.5)	5.5 (1.0–9.0)	0.018

*HSR* hypersensitivity reaction, *L-OHP* oxaliplatin, *Dex* dexamethasone

### Incidence of hypersensitivity reaction

**Fig. 2:**
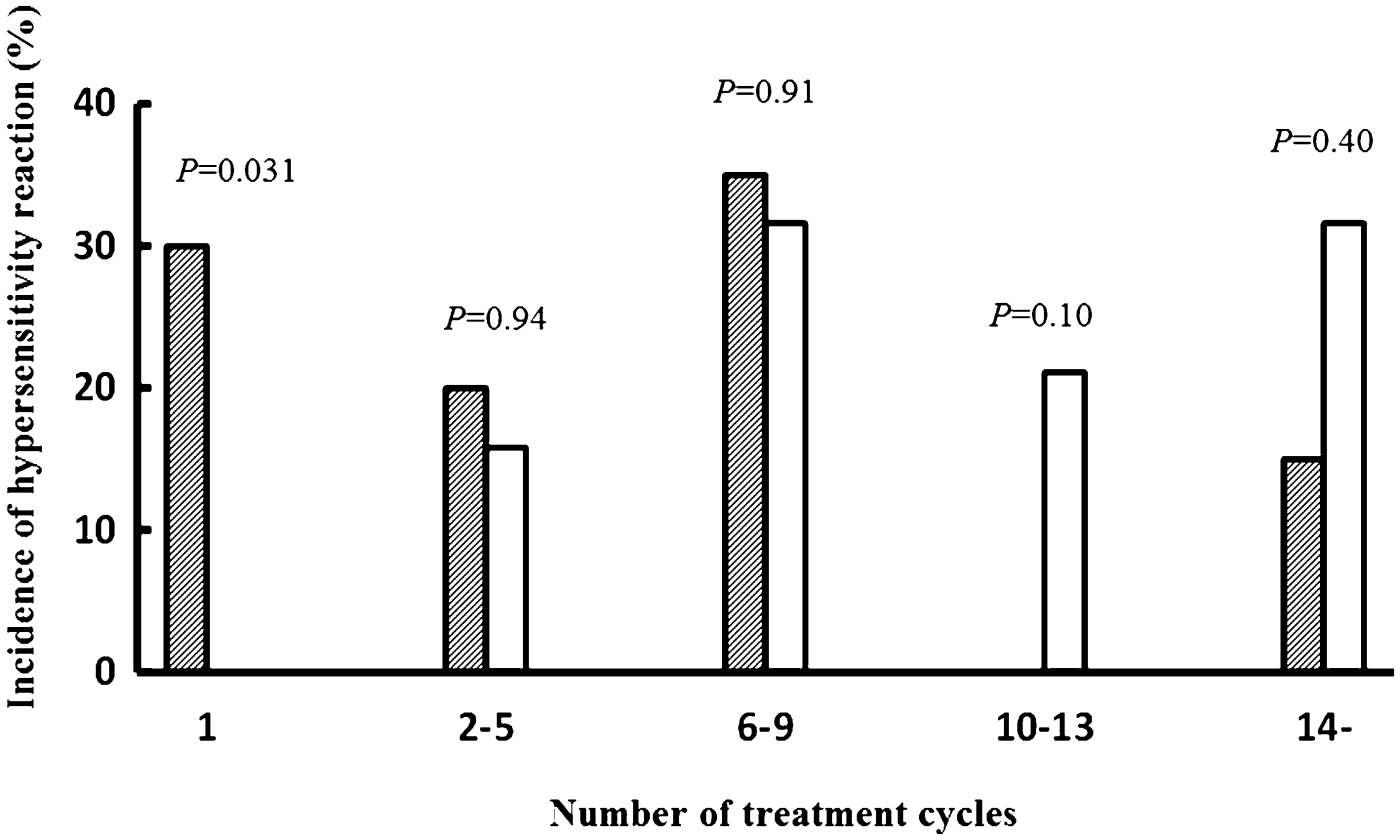
Incidence of hypersensitivity reaction in mFOLFOX6 and XELOX therapy groups. Each data was expressed hypersensitivity reaction ratio. *White bar* was expressed mFOLFOX6 therapy group. *Right hatched bar* was expressed XELOX therapy group. *P* values calculated on Chi-squared test

The relationship between the number of treatment cycles and HSRs was compared between the treatment groups. Comparisons of the first cycle and the four subsequent cycles indicated significantly more HSR occurrences during the first cycle of XELOX therapy than those during the first cycle of mFOLFOX therapy (Fig. 2). Of six patients who showed HSRs in the first cycle, one patient had a history of L-OHP administration, whereas the other five had never received L-OHP. Moreover, no differences in age, sex, or body surface area were identified between those who developed HSRs in the first cycle and those who did not (data not shown). In intergroup comparisons, 85% of HSR events in the XELOX therapy group occurred before cycle No. 10, whereas 52.7% occurred at or after cycle No. 10 in the mFOLFOX therapy group, showing a difference in the timing of HSR occurrence (*P* = 0.031). Finally, kinetic analyses of cumulative dosages (mg/m^2^ or mg) revealed no significant differences between the two groups (data not shown).

### Severity of HSR

Incidence rates of grade-1, grade-2 and higher grade HSRs were compared between those who received Dex co-infusions with L-OHP and those who did not. Incidence rates of grade-2 and higher grade HSRs were lower in patients who received Dex co-infusion (36.4%) as compared to that in patients who did not (85.7%; *P* = 0.0072).

The median cumulative dosage of L-OHP administered to patients who experienced HSRs was 628.6 (mg/m^2^). Thus, we divided these patients into two subgroups according to whether or not the cumulative dosage of L-OHP was less or more than 600 mg/m^2^ and compared incidence rates of grade-1, grade-2 and higher grade HSR. The incidence of ≥grade-2 HSRs was significantly (*P* = 0.025) less among those treated with <600-mg/m^2^ L-OHP (52.6%) than in those treated with ≥600-mg/m^2^ L-OHP (90.0%).

## Discussion

The present analyses showed no differences in HSR incidence between patients who received mFOLFOX6 (14.0%) and XELOX (19.2%) therapies, although HSR incidence rates in the present study were somewhat lower than those in previous reports of FOLFOX therapy (17.0–23.8%) [7–10, 13]. In a smaller study [14], HSR incidence rates were 16.7 and 15.0% in subjects who received XELOX and XELOX+ bevacizumab therapies, respectively. These rates were slightly lower than those observed in the present XELOX-treated cohort. However, the reported L-OHP HSR rates have tended to range widely from 8.9 to 23.8% in previous studies [5–17], which included patients with different treatment backgrounds. Therefore, we assume that the present HSR rates are within the expected range.

In previous analyses of potential risk factors for HSR occurrence, female sex, young age, and initial treatment with platinum-based anticancer agents were identified as risk factors by Parel et al. [16] and repeated L-OHP administration was identified as risk factors by Shao et al. [11]; however, these risk factors did not show any appreciable effects in the present study. This discrepancy may reflect differences in study conditions, including study periods (2004–2011 and 2006–2007, respectively), and different anticancer and adjuvant therapies for different cancer types. In addition, racial difference between the study populations may have affected the results. Sugihara et al. [20] compared rates of adverse events due to FOLFOX4 therapy in two Asian studies and four Western studies and found no racial differences in HSR incidence between the two; however, they did not perform a risk factor analysis. Subjects in the study by Shao et al. [11] were ethnically closer to the present study subjects, and their sex and age were not predictive of HSRs. Moreover, in a previous Japanese study, Shibata et al. [9] investigated HSR occurrence in 125 Japanese patients with colorectal cancer who were treated with FOLFOX4, FOLFOX6 or mFOLFOX6 therapy during 2005–2006. These authors also reported no differences in sex or age between patients who experienced HSRs and those who did not. In contrast, in 108 Japanese colorectal cancer patients who were treated with FOLFOX4 and/or mFOLFOX6 therapy during 2005–2009, Seki et al. [13] reported higher proportions of women and past allergic history among those with grade-1/2 HSRs than among those who did not suffer grade-1/2 HSR. However, our multivariate analyses may more accurately reflect the effects of colorectal cancer treatments on HSRs in the therapies that currently used frequently.

Although we compared mFOLFOX6 and XELOX therapies in colorectal cancer patients, differences in treatment methods were not related to potential risk factors for HSRs. Therefore, differences in treatment methods were not explanatory of HSR. Moreover, age, metastasis, co-infusion of Dex with L-OHP and albumin and AST levels, which were significant or near-significant factors in group comparisons of patient backgrounds, were not identified as significant risk factors. Therefore, differences in patient backgrounds were unlikely to affect HSR comparisons between the two groups. Furthermore, our multivariate analyses identified body surface area ≥1.57 m^2^ as the only significant risk factor, with an odds ratio of 2.18. This body surface area was chosen as the threshold because there were no extreme outliers among these values and the median was considered to be an appropriate statistic. In addition, cumulative L-OHP dosages were excluded from risk factor analyses as both relative (mg/m^2^) and absolute (mg) values, which indicates that differences in L-OHP dosages relative to body surface area are highly unlikely to affect HSR incidence.

When comparing subjects with HSR, the median timing of HSR onset was 10.0 cycles in the mFOLFOX group and 5.5 cycles in the XELOX group, which implies a significant difference. Alternatively, further analyses showed that HSRs occurred before cycle 10 in 85.0% of patients who had HSRs during XELOX therapy, but only in 52.7% of FOLFOX-treated patients after cycle 10. Accordingly, in Japanese FOLFOX-treated patients, HSRs occurred at 9 (4–16) cycles [7] and 9 (2–15) cycles [9], grade-1/2 HSRs occurred at 8.4 ± 4.4 cycles and grade-3/4 HSRs occurred at 9.3 ± 3.9 cycles [13]. In addition, using data from 156 and 17 patients who experienced HSRs during FOLFOX and XELOX therapies, respectively, Lee et al. [21] reported that HSRs occurred at earlier cycles in the latter group of patients (6.6 ± 0.3 versus 3.1 ± 0.6 cycles). Although HSRs occurred earlier in their FOLFOX therapy group as compared to that in our study, the observed earlier occurrence of HSRs in the XELOX therapy group is consistent with our results. In addition, no correlation between cumulative dosage (mg/m^2^ or mg) and HSR incidence was identified in the present study, which suggests that the present differences in the cycle of HSR outbreak between the two groups are caused by the difference of L-OHP dosages per one cycle between the two groups. However, the high incidence of HSRs during the first cycle of XELOX therapy warrants further investigation. In particular, HSRs to individual platinum-based compounds occur at different times and via different mechanisms [4], but reportedly involve formation of platinum-sensitive IgE antibodies as a common pathogenic mechanism [22–24]. In addition, demonstrated mechanisms underlying type I allergies to carboplatin are numerous [25], and markedly increased IgE levels were shown in a case report of patients with HSRs to L-OHP [26]. L-OHP has also been shown to act as a superantigen that induces IgE production in mononuclear cells [27]. Accordingly, HSRs to L-OHP is regarded as an acute reaction that occurs after repeat administration [18], typically after six cycles [4, 23], rather than at treatment initiation. However, the present data warrant attention to the cumulative L-OHP dosage and indicate that HSRs can occur during the first cycle of regimens with high L-OHP dosages, such as that in XELOX therapy.

On further analyses, high rates of grade-2 or higher grade HSRs were observed when Dex was not co-infused with L-OHP and when cumulative L-OHP dosages were ≥600 mg/m^2^. Because L-OHP causes vascular pain due to its acidic pH, Yoshida et al. [28] added Dex to L-OHP infusions for raising the pH during XELOX therapy, which reduced vascular pain because L-OHP causes vascular pain due to its acidic pH. Hence, the co-infusion method is now widely used and Yoshida et al. [19] showed significant decreases in HSR incidence following two co-injections of a total of 13.2 mg Dex, before and after L-OHP administration during XELOX therapy. These authors described the possibility that a change in pH caused by co-infusion of Dex could influence histamine release as an inhibitory mechanism. Although the total dosage in the present study (6.6 mg) was half of their dosages, the incidence of grade-2 and higher grade HSRs was lower after co-infusion of 1.65 mg of Dex than that when Dex was not co-infused. As the conditions for Dex used in our regimen differed greatly from those in Yoshida et al.’s regimen, we were unable to reach a conclusion on the actual inhibitory mechanism. The incidence of HSRs at grade-2 and higher was completely inhibited in Yoshida et al.’s regimen, but this was not the case for the regimen used in the present study. Identification of this inhibitory mechanism by future studies would be beneficial for the prevention of HSRs. Finally, Kidera et al. [12] compared HSR incidence between FOLFOX6-treated patients who received 6.6 mg of Dex in all courses and those who received Dex for first five courses followed by oral treatment with 50 mg diphenhydramine, 16.5 mg Dex and 20 mg famotidine from the sixth course. The HSR rate was lower in the latter group (20 versus 7%). This effect could not be investigated in the present study because no subjects were administered H1 or H2 blockers.

The present study was limited as a retrospective survey of medical records. Data related to HSRs were collected from descriptions in the medical records. The study only included colon and rectal cancer; this was to restrict the range of diagnoses and treatments across departments and thus to minimize any bias due to differences between staff involved in the treatment. Moreover, although 240 patients were included in analyses, HSRs occurred in only 16.3% of subjects, thus limiting subgroup analyses of those who experienced HSRs. However, in the current condition of constantly changing treatment methods, larger sample sizes are unlikely and warrant multicentre studies to confirm the present conclusions. However, even this will have limitations; ideally, a prospective multicentre study should be conducted within a defined period of time.

Our findings indicate that XELOX therapy with high L-OHP dosages should be administered with attention to cumulative L-OHP dosages and possible occurrence of HSRs in the first cycle. The present data also suggests that grade-2 and higher grade HSRs may be reduced by co-infusion of Dex with L-OHP. Finally, rates of ≥grade-2 HSRs are increased when cumulative L-OHP dosages exceed 600 mg/m^2^.

## References

[1] de Gramont A, Figer A, Seymour M (2000). Leucovorin and fluorouracil with or without oxaliplatin as first-line treatment in advanced colorectal cancer. J Clin Oncol.

[2] National Comprehensive Cancer Network (NCCN) Clinical Practice Guidelines in Oncology. Colon/Rectal Cancer, 2015 ver.2. https://www.nccn.org/professionals/physician_gls/f_guidelines.asp. Accessed 2 Dec 2016

[3] Watanabe T, Itabashi M, Shimada Y (2015). Japanese Society for Cancer of the Colon and Rectum (JSCCR) Guidelines 2014 for treatment of colorectal cancer. Int J Clin Oncol.

[4] Makrilia N, Syrigou E, Kaklamanos I (2010). Hypersensitivity reactions associated with platinum antineoplastic agents: a systematic review. Met Based Drugs.

[5] Brandi G, Pantaleo MA, Galli C (2003). Hypersensitivity reactions related to oxaliplatin (OHP). Br J Cancer.

[6] Andre T, Boni C, Mounedji-Boudiaf L (2004). Oxaliplatin, fluorouracil, and leucovorin as adjuvant treatment for colon cancer. N Engl J Med.

[7] Suenaga M, Mizunuma N, Shinozaki E (2008). Management of allergic reactions to oxaliplatin in colorectal cancer patients. J Support Oncol.

[8] Ichikawa Y, Goto A, Hirokawa S (2009). Allergic reactions to oxaliplatin in a single institute in Japan. Jpn J Clin Oncol.

[9] Shibata Y, Ariyama H, Baba E (2009). Oxaliplatin-induced allergic reaction in patients with colorectal cancer in Japan. Int J Clin Oncol.

[10] Mori Y, Nishimura T, Kitano T (2010). Oxaliplatin-free interval as a risk factor for hypersensitivity reaction among colorectal cancer patients treated with FOLFOX. Oncology.

[11] Shao YY, Hu FC, Liang JT (2010). Characteristics and risk factors of oxaliplatin-related hypersensitivity reactions. J Formos Med Assoc.

[12] Kidera Y, Satoh T, Ueda S (2011). High-dose dexamethasone plus antihistamine prevents colorectal cancer patients treated with modified FOLFOX6 from hypersensitivity reactions induced by oxaliplatin. Int J Clin Oncol.

[13] Seki K, Senzaki K, Tsuduki Y (2011). Risk factors for oxaliplatin-induced hypersensitivity reactions in Japanese patients with advanced colorectal cancer. Int J Med Sci.

[14] Arii D, Ikeno Y, Murooka K (2012). Expression of allergic reactions to oxaliplatin. Jpn J Cancer Chemother.

[15] Yamauchi H, Goto T, Takayoshi K (2015). A retrospective analysis of the risk factors for allergic reactions induced by the administration of oxaliplatin. Eur J Cancer Care.

[16] Parel M, Ranchon F, Nosbaum A et al (2014) Hypersensitivity to oxaliplatin: clinical features and risk factors. BMC. Pharmacol Toxicol 15:1. http://www.biomedcentral.com/2050-6511/15/1 10.1186/2050-6511-15-1 PMC3896838 24417770

[17] Park HJ, Lee JH, Kim SR (2016). A new practical desensitization protocol for oxaliplatin-induced immediate hypersensitivity reactions: a necessary and useful approach. J Investig Allergol Clin Immunol.

[18] Aroldi F, Prochilo T, Bertocchi P (2015). Oxaliplatin-induced hypersensitivity reaction: underlying mechanisms and management. J Chemther.

[19] Yoshida Y, Hirata K, Matsuoka H (2015). A single-arm Phase II validation study of preventing oxaliplatin-induced hypersensitivity reactions by dexamethasone: the AVOID trial. Drug Des Devel Ther.

[20] Sugihara K, Ohtsu A, Shimada Y (2012). Safety analysis of FOLFOX4 treatment in colorectal cancer patients: a comparison between two Asian studies and four western studies. Clin Colorectal Cancer.

[21] Lee S, Kang H, Song W (2014). Overcoming oxaliplatin hypersensitivity: different strategies are needed according to the severity and previous exposure. Cancer Chemther Pharmacol.

[22] Leguy-Seguin V, Jolimoy G, Coudert B (2007). Diagnostic and predictive value of skin testing in platinum salt hypersensitivity. J Allergy Clin Immunol.

[23] Lenz HJ (2007). Management and preparedness for infusion and hypersensitivity reactions. Oncologist.

[24] Bano N, Najam R, Qazi F (2016). Clinical features of oxaliplatin induced hypersensitivity reactions and therapeutic approaches. Asian Pacific J Cancer Prevent.

[25] Iwamoto T, Yuta A, Tabata T (2012). Evaluation of basophil CD203c as a predictor of carboplatin-related hypersensitivity reaction in patients with gynecologic cancer. Biol Pharm Bull.

[26] Kitada N, Dan T, Takara K (2007). Oxaliplatin-induced hypersensitivity reaction displaying marked elevation of immunoglobulin E. J Oncol Pharm Pract.

[27] Syrigou E, Syrigos K, Saif MV (2008). Hypersensitivity reactions to oxaliplatin and other antineoplastic agents. Curr Allergy Asthma Rep.

[28] Yoshida Y, Hoshino S, Aisu N (2012). Dexamethasone as a means not only for controlling vascular pain caused by the administration of oxaliplatin via the peripheral vein but also for controlling oxaliplatin-induced hypersensitivity reactions. Br J Med Med Res.

